# Leveraging an immune cell signature to improve the survival and immunotherapy response of lung adenocarcinoma

**DOI:** 10.7150/jca.90515

**Published:** 2024-01-01

**Authors:** Jiacheng Zhang, Tianrui Kuang, Keshuai Dong, Jia Yu, Weixing Wang

**Affiliations:** 1 Department of Hepatobiliary Surgery, Renmin Hospital of Wuhan University, Wuhan, 430060, Hubei Province, People's Republic of China.; 2 Central Laboratory, Renmin Hospital of Wuhan University, Wuhan, 430060, Hubei Province, People's Republic of China.; 3 Department of General Surgery, Renmin Hospital of Wuhan University, Wuhan, 430060, Hubei Province, People's Republic of China.

**Keywords:** immune cell, immunotherapy, lung adenocarcinoma, prognosis, tumor microenvironment

## Abstract

**Background:** Immune cells play a critical role in the prognosis of cancer. However, the function of different immune cell types in lung adenocarcinoma (LUAD) and the development of a prognostic signature based on immune cell types have not been comprehensively investigated.

**Methods:** We collected and included a total of 2499 LUAD patients and performed calculations to determine the penetration level of 24 immune cells. This examination was conducted using the macro-gene-based approach provided by ImmuCellAI. We performed a meta-analysis using Lasso-Cox analysis to establish the immune cell pair score (ICPS). We conducted a survival analysis to measure differences in survival across ICPS-risk groups. Wilcox test was used to measure the difference in expression level. Spearman correlation analysis was used for the relevance assessment.

**Results:** We collected a total of 24 immune cell types to construct cell pairs. Utilizing 17 immune cell pairs, we constructed and validated the ICPS, which plays a critical role in stratifying survival and dynamically monitoring the effectiveness of immunotherapy. Additionally, we identified several candidate drugs that target ICPS.

**Conclusions:** The ICPS shows promise as a valuable tool for identifying suitable candidates for immunotherapy among patients. Our comprehensive assessment of immune cell interactions in LUAD contributes to a deeper understanding of infiltration patterns and functions, thereby guiding the development of more efficacious immunotherapy strategies.

## Introduction

Throughout the world, lung cancer is one of the most prevalent malignant tumors that pose a significant risk to human health. This disease has the highest incidence rate as well as mortality rate. Lung adenocarcinoma (LUAD) is the most prevalent histological subtype of lung cancer, possessing unique biological characteristics 1-3. Clinically, patients with lung adenocarcinoma often lack typical clinical symptoms or even have no symptoms in the early stage, are prone to distant metastasis, and have high drug resistance. These characteristics also make the clinical treatment of lung adenocarcinoma a great challenge. Recently, with the rapid development of medical molecular biology, the treatment of LUAD has gradually diversified, and its pathological classification is also gradually refined. Lung cancer is moving towards the stage of precise diagnosis and treatment. However, the emergence of new treatment modes has also brought new problems and challenges to the clinic. The era of precision medicine has put forward higher requirements for researchers and medical staff.

With the deepening of research, researchers have gradually realized that the continuous and dynamic interaction between cancer cells and the tumor microenvironment (TME) is the key factor in promoting tumor occurrence, development, and metastasis 4. It is composed of several different types of cells and secreted factors that trigger tumor growth. The important biological characteristics of the tumor tissue microenvironment are tissue hypoxia, low pH, increased tissue stiffness, nutrient deprivation, the formation of interstitial hypertension, and immune-inflammatory reactions 5, 6. The TME can play an important regulatory role in tumor cell proliferation, survival, tumor angiogenesis, self-renewal of tumor stem cells, and tumor invasion and metastasis by providing growth factors, cell survival-promoting factors, extracellular matrix, and numerous adhesion molecules for tumor cells 7. Among them, the tumor immune microenvironment, which is composed of macrophages, dendritic cells, neutrophils, B cells, T cells, tumor-associated fibroblasts, and secreted cytokines, constructs the tumor immune barrier, thereby affecting the response rate of immunotherapy 8. The rapid development of immunotherapy has brought significant development opportunities for tumor therapy in recent years. Immune checkpoint blockers can improve anti-tumor immune response by regulating T cell activity, which has become one of the current research hotspots and the most promising strategies.

In this study, we measured immune cell infiltration levels within each cohort of samples using meta-multiple LUAD cohorts. We collected 24 types of immune cells from ImmuCellAI and established the immune cell pair (ICP) to understand the complex interactions between immune cells realistically. Finally, we created and validated the immune cell pair score (ICPS), which showed strong predictive ability in predicting prognosis and evaluating the efficacy of immunotherapy in LUAD.

## Materials and Methods

### Data resources

The transcriptome cohorts of LUAD were collected from multiple public databases including the Cancer Genome Atlas (TCGA) databases (TCGA-LUAD), and the gene expression omnibus (GEO) database (n=13). Besides, the immunotherapy data IMvigor210 was downloaded from the “IMvigor210CoreBiologies” R package containing 348 samples corresponding to clinicopathological information 9. GSE78220, including patients treated with anti-PD-1 antibody, was downloaded from the GEO database 10. After surgical removal, the pathology was clearly diagnosed as glandular cancer, and the prognostic information was complete, and patients who were not immunotherapeutic at the time of sampling were included in the study. We collected 14 LUAD cohorts 11-26 with transcriptome data and survival data (Table S1). The “DEseq.2” R package was used for the normalization and log (2+1) transformation of TCGA-LUAD data. The normalization of the IMvigor210 cohort was also conducted via “DEseq.2” R package. What's more, a LUAD-specific, meta-entire cohort was collected after preprocessing, merging, and ComBat-adjusting the 14 cohorts via the “sva” R package. The meta-entire cohort included a total of 2499 LUAD samples meeting the requirements for complete information.

### The constructed of immune cell pair score (ICPS)

Firstly, the level of immune cell infiltration was conducted by a macro-gene-based approach from the ImmuCellAI (Immune Cell Abundance Identifier) 27, 28. We can get the infiltration degree of 24 different types of immune cells in each LUAD sample. Then, to increase the comparability between cohorts from different sources and further reduce the batch effect, the immune cell pair index (ICPI) was established. The ICPI was regarded as 1 when the level of immune cell A infiltration exceeded that of another immune cell. Conversely, when the level of immune cell A was lower than that of another immune cell, the ICPI was assigned as 0. In the subsequent phase of analysis, various ICPIs exhibiting consistent values (0 or 1) were excluded to alleviate potential biases stemming from platform-specific priority measurements. Additionally, the entire meta-cohort was split into equal-sized subsets: the meta-training cohort (n=1249) and the meta-testing cohort (n=1250), maintaining a 1:1 ratio according to the random algorithm. The ICPS was constructed using the Lasso regression analysis via the “glmnet” R package in the meta-training cohort 29. We calculated the coefficients for each ICP using the multivariate Cox proportional hazards model.



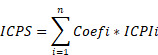



Based on the above formula, we could get the ICPS of each LUAD sample. We employed a time-dependent receiver operating characteristic (ROC) curve analysis to effectively classify patients with LUAD into high- and low-risk groups via the "survivalROC" R package 30. Subsequently, the threshold value for classification in this study was determined based on the ICPS value that exhibited the minimal deviation from the ROC curve at a specific point. As a result, patients with LUAD from various cohorts, including TCGA-LUAD, training, testing, and meta-cohort, were classified into two risk groups according to their ICPS risk level.

### Association between ICPS and clinical features of LUAD patients

To explore the prognostic significance of the ICPS, we utilized the "survival" R package to perform subsequent survival analyses. To assess the survival differences between high-risk and low-risk groups based on ICPS, we partitioned the meta-entire cohort into training and testing cohorts. To validate the prognostic role of ICPS, we also utilized the entire pooled cohort. The log-rank test was utilized to assess the significance of survival differences between the groups, considering P < 0.05 as statistically significant. In addition to analyzing ICPS, we also carefully examined other clinical indicators such as age, gender, and TNM staging to evaluate the differences between the risk groups. Statistical significance was established if the p-value obtained from the chi-square test was below the threshold of 0.05. This analysis aided in identifying statistically significant differences in clinical indicators between the low-risk and high-risk cohorts.

### Association between ICPS and several mutation Indices

Firstly, we explored the mutation landscape of different risk groups with LUAD via the “maftools” R package 31. Next, the single-nucleotide variant (SNV) data of LUAD was downloaded from the TCGA database. Based on the SNV data, the tumor mutation burden (TMB) was evaluated to analyze the differences between the ICPS groups 32. Besides, we analyzed the differences in tumor stemness indicators between ICPS groups, such as transcriptomic signatures based-index (mRNAsi), DNA methylation based-index (mDNAsi), epigenetic regulation based-index (EREG-mDNAsi), differentially methylated probes-based stemness index (DMPsi), and enhancer-based stemness index (ENHsi). The tumor stemness was closely related to tumor prognosis based on the previous studies. What's more, we compared the genomic instability state between ICPS groups according to the homologous recombination deficit (HRD) score, which could help predict the responsiveness of malignant tumors to platinum chemotherapy and PARPi therapy. The Wilcoxon test was used to compare the group differences mentioned above.

### The immune landscape of ICPS in LUAD

Firstly, the level of tumor immune infiltrating cells of each LUAD sample was obtained based on the TIMER 33, 34 (https://cistrome.shinyapps.io/timer/), EPIC 35, CIBERSORT 36, and MCPcounter 37. We explored the relationship between ICPS and tumor immune infiltrating cells by Pearson's correlation analysis. Next, the immune score, stromal score, and tumor purity of the TCGA-LUAD cohort were calculated to explore the association between ICPS and the immune landscape via the “ESTIMATE” algorithm. The T cell dysfunction and exclusion (TIDE) method 38, 39 was utilized to investigate the response to different immune treatments. Additionally, a quantitative assessment was conducted on LUAD samples. The TIDE scores of the LUAD samples were obtained from the website: http://tide.dfci.harvard.edu/. The previous study identified 49 molecular markers related to immune characteristics. Building upon the above-mentioned study, we examined the correlation between ICPS and tumor immune characteristics. Given the substantial importance of immune checkpoints (ICS) and immunogenic cell death (ICD) modulators in tumor immunity, our objective was to investigate the correlation between ICPS and both ICS and ICD modulators. Correlation statistical analyses were performed using the Spearman method. Furthermore, we measured 29 immune functions in the ICPS risk groups via the "CIBERSORT" R package. Subsequent analysis was performed to examine the disparities in immune function across the groups.

### Investigating the role of ICPPI in response to immunotherapy

The IMvigor210 cohort including 298 patients who were treated with anti-PD-L1 immunotherapy and GSE78220, including patients treated with anti-PD-1 antibody were used to investigate the association between the ICPS and immunotherapy after normalizing. The Kruskal-Wallis test was used to investigate the difference in ICPS scores across the various response groups (CR, PR, PD, and SD). The “survival” R package was used to explore the difference in survival between the ICPS risk groups. The ROC curves were used to assess the prediction of the efficacy of immunotherapy via the “pROC” R package.

### Drug sensitivity exploring

Subsequently, our efforts focused on identifying numerous innovative therapeutic drugs, which provide several new options for treating LUAD. We initially identified the differentially expressed genes (DEGs) between the low-risk and high-risk ICPS groups within the entire meta-cohort via the "limma" R package. Next, we screened for the overlap of up-regulated and down-regulated DEGs between the ICPS-related DEGs and normal-ICPS-related DEGs to perform Connectivity map (CMap) analysis in CLUE (https://clue.io/). Small molecule drugs with |score| ≥ 90 were regarded as potential drugs.

### Statistical Analysis

All statistical analyses were conducted using the R software (version 4.1.0). The threshold for statistical significance was set as P < 0.05.

## Results

### Analytic Pipeline

**Figure 1:**
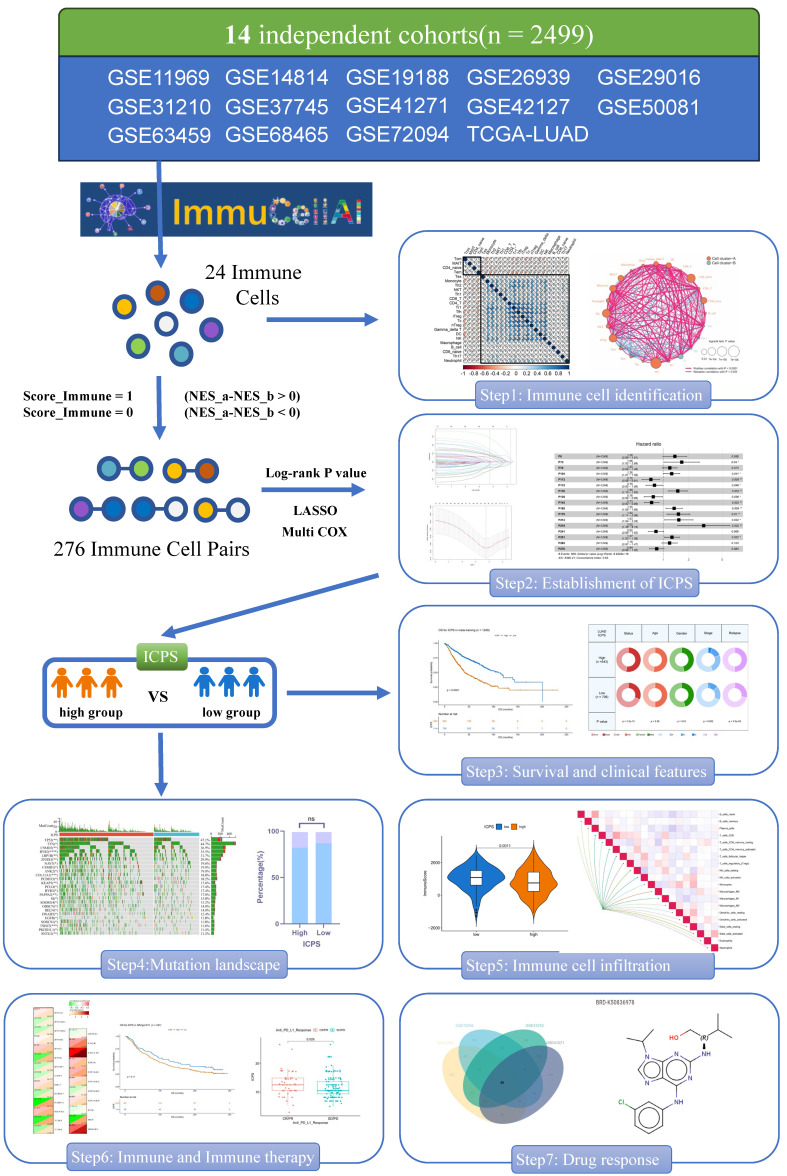
The flow diagram of this study.

The LUAD data were obtained from the TCGA and GEO databases. The data were subjected to quality control, batch effect removal, normalization, organization of mutation, and clinical, and survival data, before being used for subsequent analysis. Figure 1 illustrated the main analysis process of this study.

### ICPS Construction

**Figure 2:**
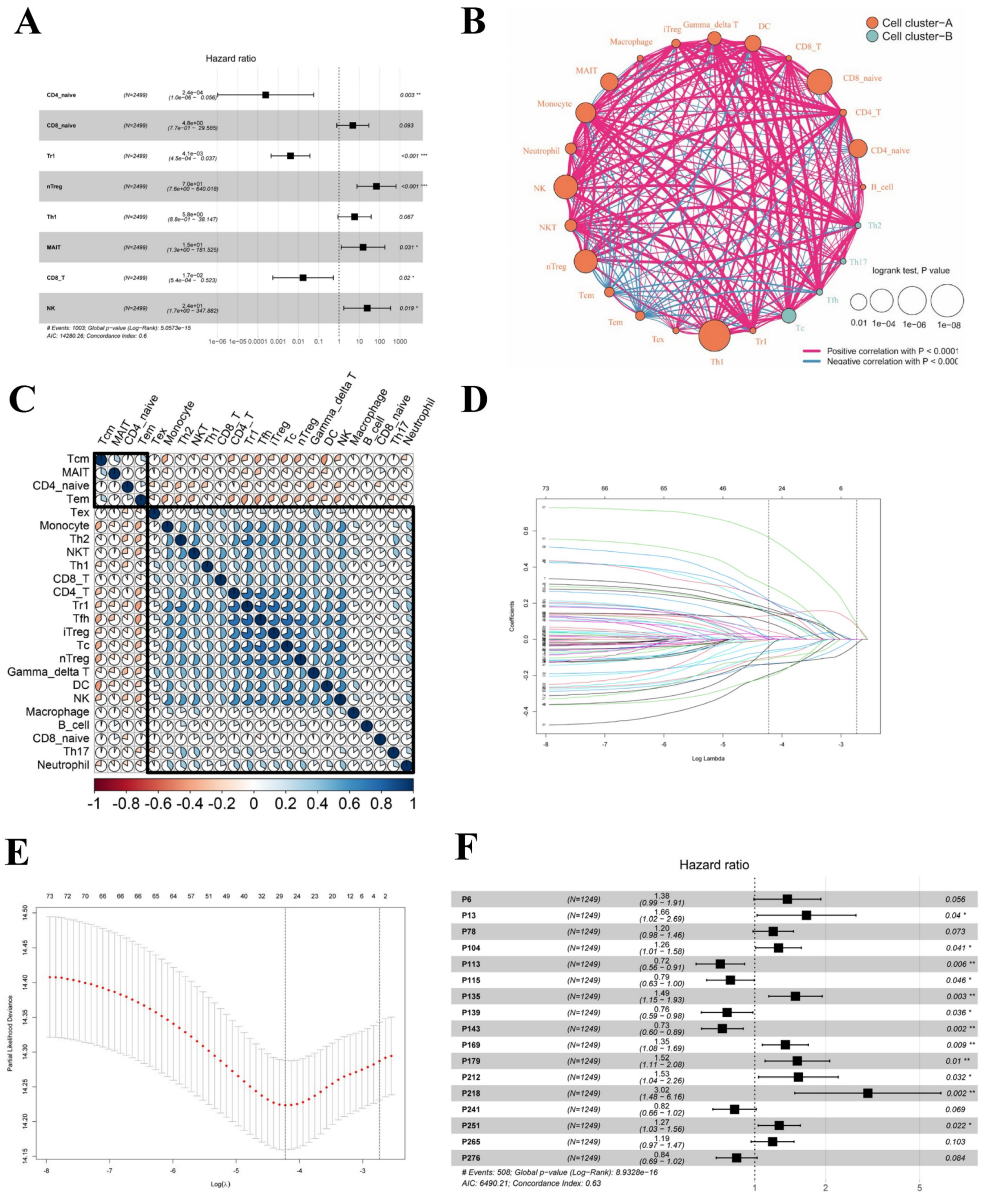
Screening of immune cells and establishment of immune cell pair score (ICPS). (A) Forest plot for the Hazard Ratios (HRs) of Immune cells. (B, C) Cellular interaction and survival landscape of the 24 immune cell types. (D, E) Plot of partial likelihood deviance for the 17 immune cell pairs (ICPs) associated with survival in the training set. (F) Forest plot for the HRs of ICPs used for ICPS construction.

**Figure 3:**
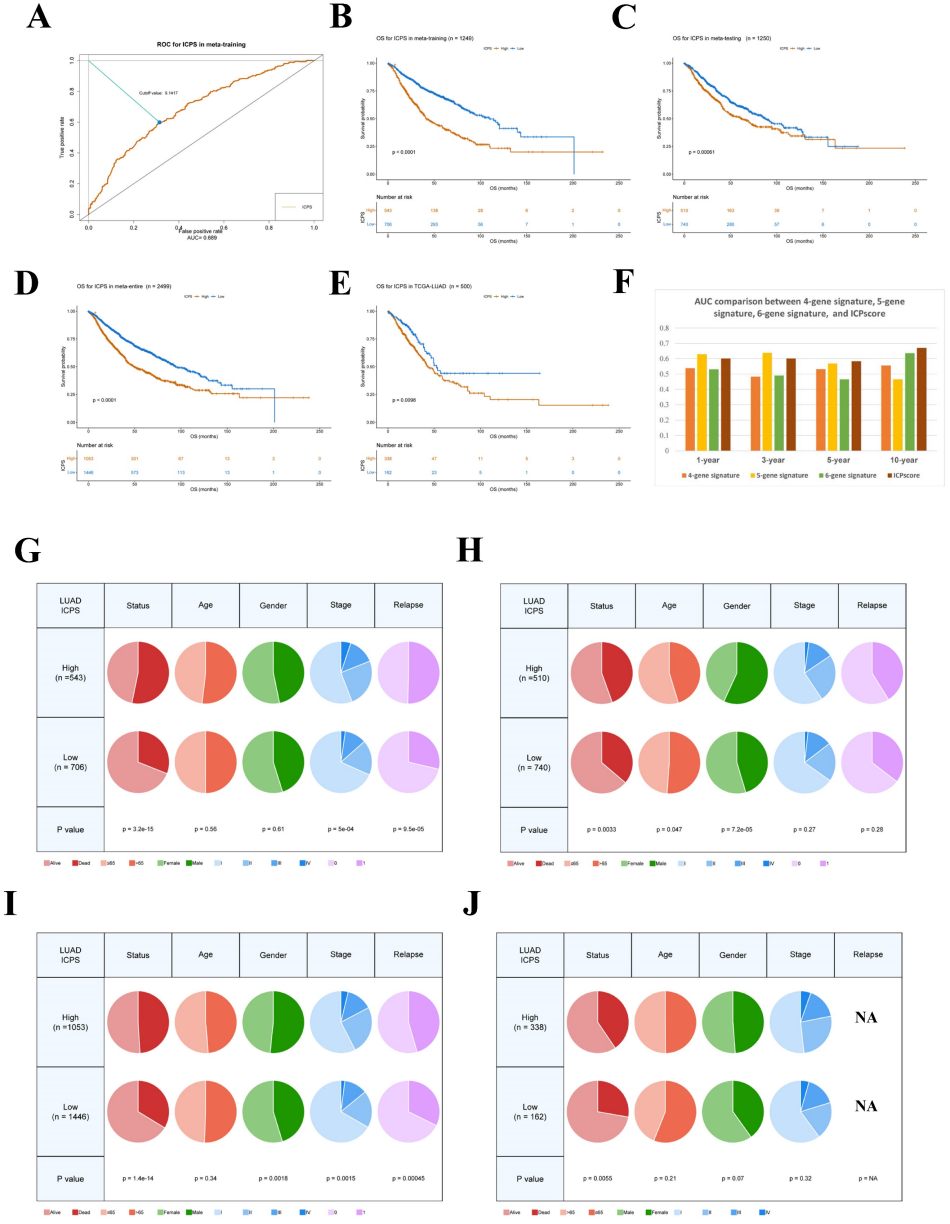
Survival and clinical difference between ICPS high-risk and ICPS low-risk group. (A) Time-dependent ROC curve for ICPS in the meta-training cohort at 5 years. (B) Overall survival curve for ICPS in the training cohort (n=1249). (C) Overall survival curve for ICPS in the testing cohort (n=1250). (D)Overall survival curve for ICPS in the entire meta-cohort(n=2499). (E) Overall survival curve for ICPS in the TCGA-LUAD data (n=500). (F) AUC comparison between 4-gene signature, 5-gene signature, 6-gene signature, and ICPS. (G-J) The differences in clinical features including status, age, gender, stage, and relapse between the two ICPS risk groups. (G) meta-training cohort, (H) meta-testing cohort, (I) entire meta-cohort, (J) TCGA-LUAD cohort.

Initially, the ImmuCellAI was employed to evaluate immune cell infiltration of the 24 different types in each sample (**Table S2**). Univariate Cox analysis was conducted to determine the prognostic significance of the 24 distinct immune cell types (**Table S3**). **Figures 2B and 2C** illustrated the complex connections between the 24 types of immune cells in LUAD, with a majority of them showing positive connections. In contrast, central memory T cell (Tcm) and effector memory T cell (Tem) exhibited a strong negative connection with other cells. The results suggested that these distinct immune cells could have varying roles in immune infiltration, either working synergistically or antagonistically. Specifically, naïve CD4^+^ T cell, tregulatory1 cell (Tr1), and CD8^+^ T cell were identified as risk factors for overall survival (OS) in LUAD. Natural Treg cell (nTreg), mucosal-associated invariant T cell (MAIT), and natural killer cell (NK) were identified as favorable factors for OS in LUAD (**Figure 2A**). As described in the methodology, a total of 276 immune cell pairs (ICPs) were established using 24 different types of immune cells (**Table S4**). The entire set of ICPs underwent a log-rank test. A total of 56 ICPs were included in the lasso regression equatio**n (Figure 2D, E**), followed by multivariate regression analysis that involved 28 ICPs (**Table S5**). Finally, 17 ICPs (**Table S6**) were selected for the construction of ICPS (**Figure 2E**). The area under the curve (AUC) value of the ROC curve was 0.689 at 5 years (**Figure 3A**). Additionally, LUAD was split into high-risk and low-risk subgroups using ICPS=9.1417 as the threshold (**Figure 3A, Table S7, Figure S1**).

### Relationship between ICPS and Clinical Features of LUAD

As mentioned earlier, the meta-cohort (n=2499) was divided into a training cohort (n=1249) and a testing cohort (n=1250). Within the training cohort, patients were stratified into two groups based on their ICPS levels: the high-risk group (n=543) and the low-risk group (n=706), according to the predetermined cutoff value. **Figure 3B** demonstrated that LUAD patients with low ICPS exhibited better OS (*P* < 0.05). The result was also validated in the testing cohort (*P* < 0.05), meta-entire cohort (*P* < 0.05), and TCGA-LUAD cohort (*P* < 0.05) (**Figure 3C-E**). Overall, patients with LUAD in the high-risk group of ICPS were at a higher risk of mortality, thus indicating ICPS as a prognostic indicator for LUAD. To determine whether the ICPS was better than previous prognostic signatures, three multiple gene signatures were collected and included in the present study. As shown in **Figure 3F,** the ICPS showed a better prognosis prediction potential compared to the four-gene signature 40, five-gene signature 41, and six-gene signature 42 in the TCGA-LUAD cohort, especially the ability to forecast over 5 and 10 years. Furthermore, we conducted further investigation into the clinical differences between the risk groups based on ICPS. Additionally, we conducted validation across four cohorts (**Figure 3G-J**). These clinical characteristics included survival status, age, gender, stage, and grade of relapse (**Table S8**). The results indicated that patients in the high-risk group of ICPS across the four cohorts exhibited higher rates of relapse (*P* < 0.05 (training cohort), *P* = 0.28(test cohort),* P* < 0.05 (total cohort)) and poorer survival status (higher proportion of mortality) (*P* < 0.05 (training cohort), *P* < 0.05 (test cohort),* P* < 0.05 (total cohort), *P* < 0.05 (TCGA-PAAD cohort)).

### Association between ICPS and Mutation

**Figure 4:**
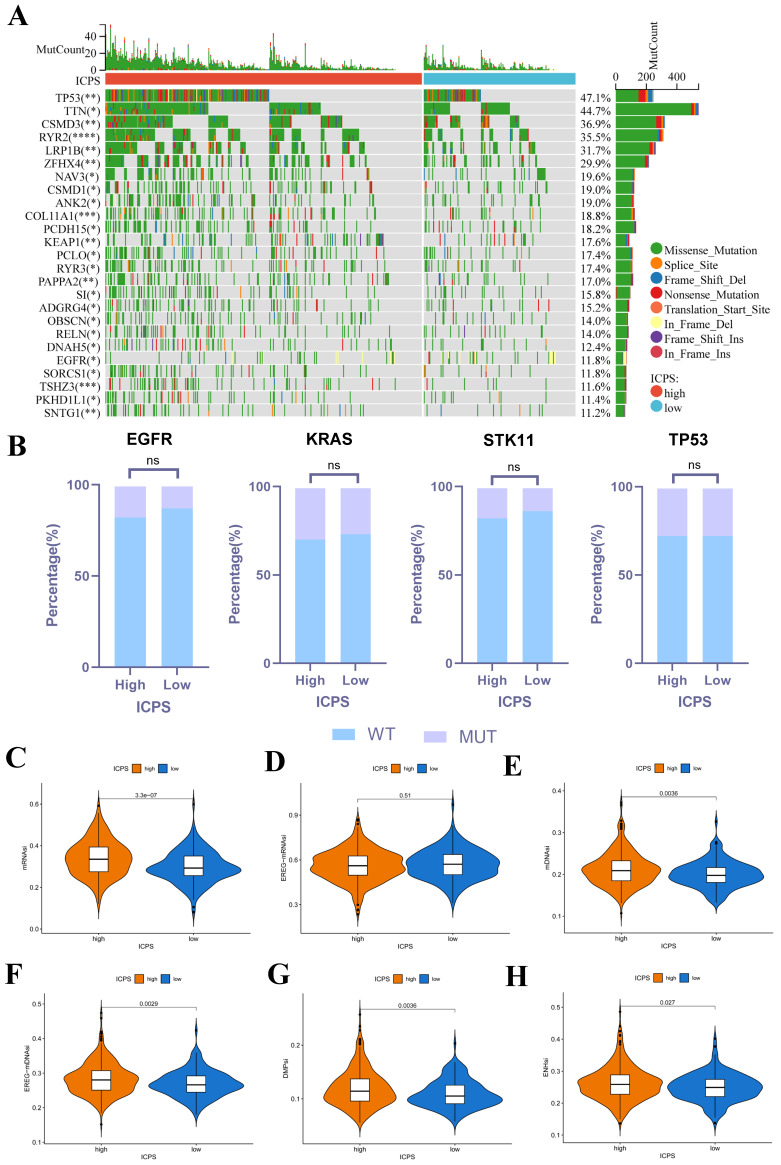
Correlation between ICPS and genomic mutations using TCGA-LUAD data. (A) The waterfall plots of the top 25 genes with the highest mutation rate in the TCGA-LUAD. (B) Correlation between ICPS and gene mutation. (C) Correlation between ICPS and mRNAsi. (D) Correlation between ICPS and EREG-mRNAsi. (E) Correlation between ICPS and mDNAsi. (F) Correlation between ICPS and EREG-mDNAsi. (G) Correlation between ICPS and DMPsi. (H) Correlation between ICPS and ENHsi.

**Figure 5:**
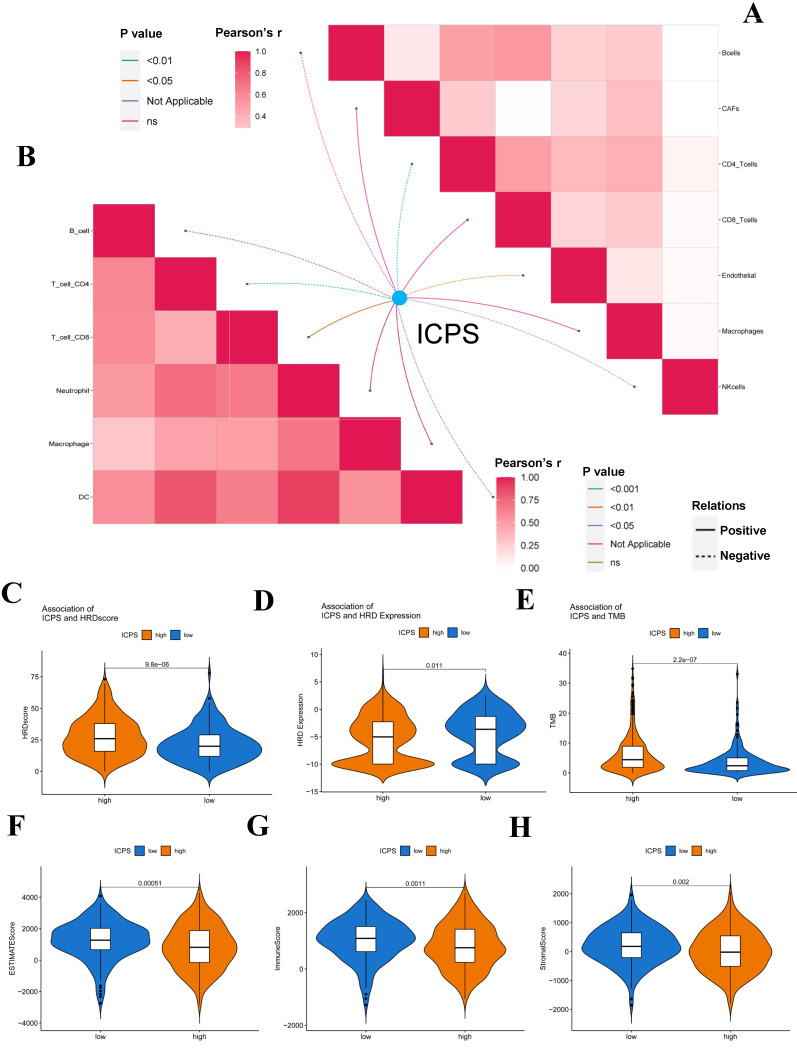
Correlation between ICPS and immune features. (A) Correlation between ICPS and immune cells in TIMER database. (B) Correlation between ICPS and immune cells based on EPIC. (C) Correlation between ICPS and HRD score. (D) Correlation between ICPS and HRD expression. (E) Correlation between ICPS and TMB. (F) Correlation between ICPS and ESTIMATE score. (G) Correlation between ICPS and immune score. (H) Correlation between ICPS and stromal score.

**Figure 4A** presented the mutation landscape of the 25 most highly mutated genes in patients with LUAD based on TCGA data. The high-risk cohort displayed a significantly higher mutation rate compared to the low-risk cohort. However, for some special mutations in LUAD, such as EGFR, KRAS, STK11, and TP53, no significant difference was found between the high and low ICPS groups, both in the wild-type and mutation-type of these genes (**Figure 4B, Table S9,** *P > 0.05*). Higher TMB, coupled with increased somatic mutation rates, has been associated with enhanced anti-cancer immunity. **Figure 5E** demonstrates that the TMB level exhibited a significant increase in the ICPS high-risk group in comparison to the low-risk group (*P < 0.05*).

RNAsi was identified as a novel predictor associated with stem-like characteristics and tumor prognosis. No significant difference was observed in EREG-mRNAsi score levels between the two groups (**Figure 4D**, *P > 0.05*). Patients in the ICPS low-risk group exhibited lower mRNAsi scores (**Figure 4C**, *P < 0.05*), mDNAsi (**Figure 4E**, *P < 0.05*), EREG-mDNAsi (**Figure 4F**, *P < 0.05*), DMPsi (**Figure 4G**, *P < 0.05*), and ENHsi (**Figure 4H**, *P < 0.05*) when compared to those in ICPS high-risk set. HRD leads to impaired repair of double-strand breaks, making it a common driver of tumorigenesis. Patients in the ICPS low-risk set showed lower HRD scores and higher HRD expression compared to those in the ICPS high-risk set (**Figure 5C-D**, *P < 0.05*) (**Table S10**).

### The immune landscape of ICPS in LUAD

**Figure 6:**
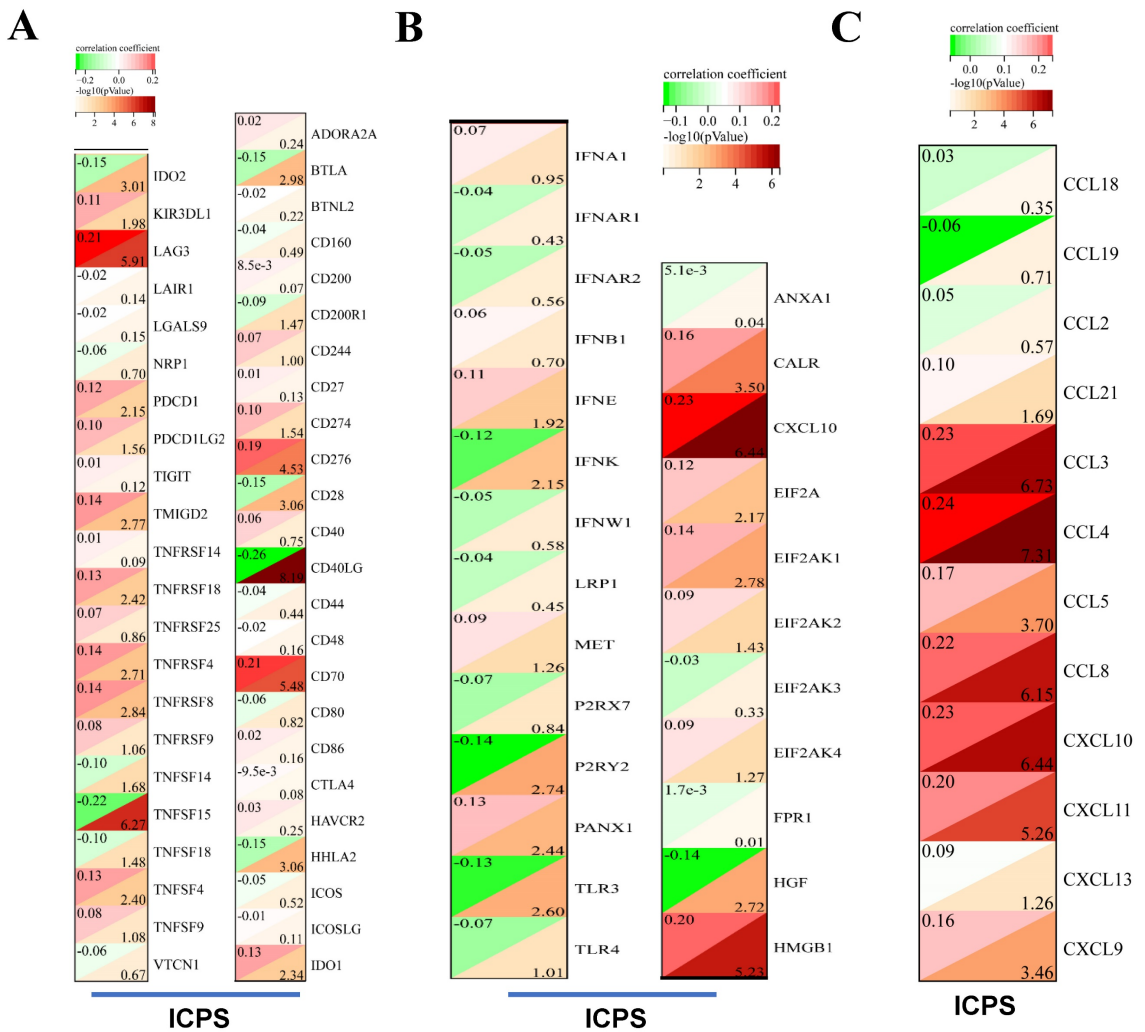
Immune landscape of ICPS in LUAD. (A) Correlation between ICPS and immune indicators. (B) Correlation between ICPS and ICD modulators. (C) Correlation between ICPS and TLSs.

The association between ICPS and tumor immune infiltrating cells was explored. The study concluded that ICPS showed positive correlations with macrophages, CD8^+^-T cells, and cancer-associated fibroblasts (CAFs). Furthermore, ICPS exhibited a negative association with NK cells, B cells, CD4^+^-T-cells, and endothelial cells, as indicated by the TIMER (**Figure 5A, Table S11**). Additional analyses demonstrated that ICPS showed positive associations with cytotoxic lymphocytes, NK cells, CD8^+^-T-cells, monocytic lineage, and fibroblasts. Conversely, ICPS demonstrated negative associations with B lineage, myeloid dendritic cells, T-cells, neutrophils, and endothelial cells from the EPIC (**Figure 6B, Table S11**). Furthermore, the results obtained using the CIBERSORT and MCPcounter algorithms were in line with the aforementioned findings (**Figure S2, Table S11**). Immune-related scores are valuable in assessing the prognosis of tumor patients and the effectiveness of immunotherapy. Our results indicated that patients in the low-risk group exhibited higher ESTIMATE scores (**Figure 5F**, *P < 0.05*), immune scores (**Figure 5G**, *P < 0.05*), and stromal scores (**Figure 5H**, *P < 0.05*) compared to those the high-risk set (**Table S10**). The analysis revealed that LAG3, PDCD1, TMIGD2, TNFRSF18, TNFRSF4, TNFRSF8, TNFSF4, CD276, CD70, and IDO1 displayed positive associations with ICPS. In contrast, IDO2, TNFSF15, BTLA, CD28, CD40LG and HHLA2 exhibited negative associations with ICPS (**Figure 6A**). Additionally, PANX1, CALR, CXCL10, EIF2A, EIF2AK1, and HMGB1 showed a positive correlation with ICPS. Conversely, IFNK, P2RY2, TLR3, and HGF exhibited a negative correlation with ICPS (**Figure 6B**). Tertiary lymphoid structures (TLS) were found to play a crucial role in tumor immunity. This study examined the correlation between ICPS and TLS gene signatures. The results revealed a positive association between ICPS and CCL3, CCL4, CCL5, CCL8, CXCL10, CXCL11, and CLCL9 (**Figure 6C**) (**Table S12**).

### Relationship between ICPS and immunotherapy

**Figure 7:**
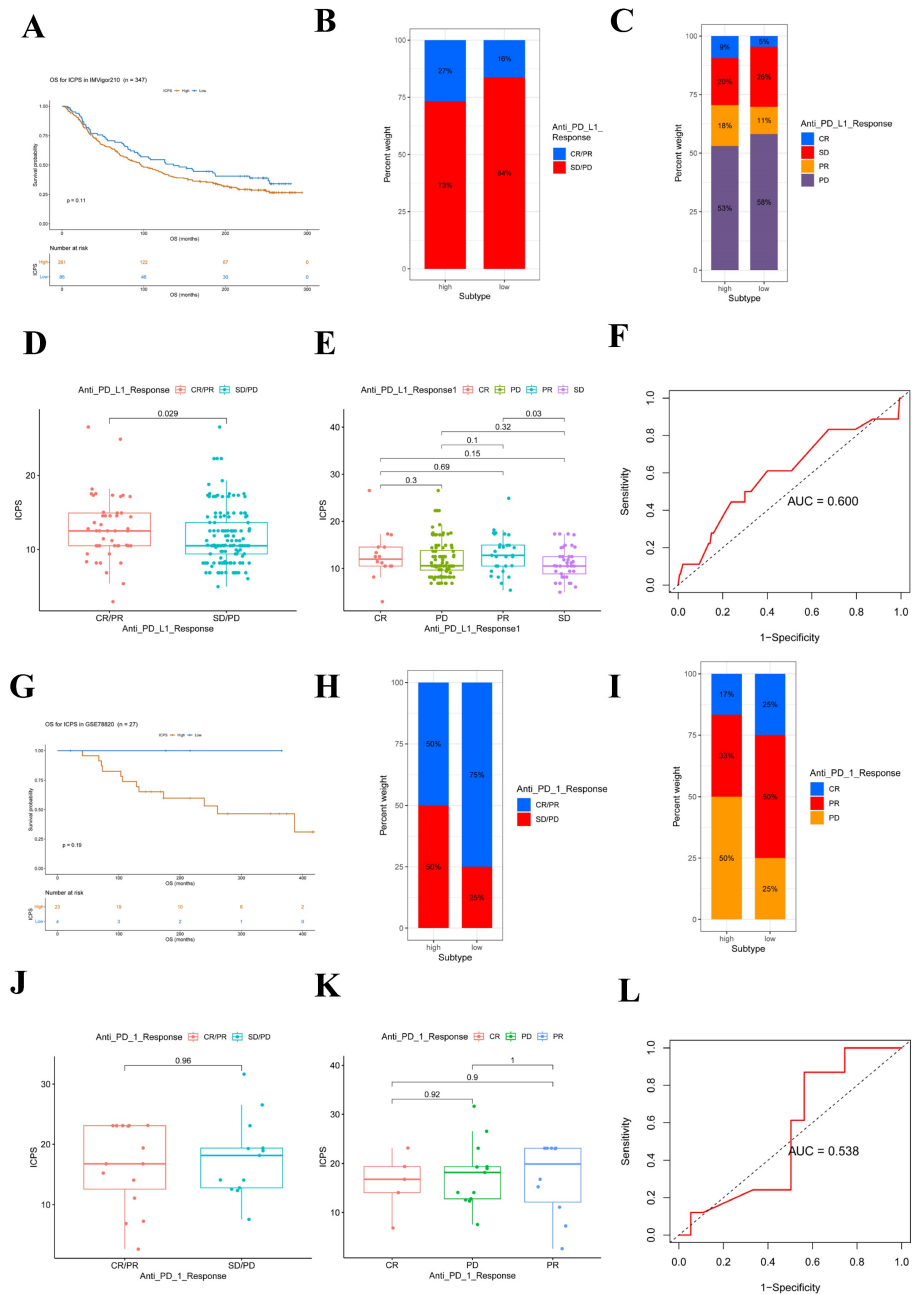
ICPS for predicting the effect of immunotherapy. (A) Kaplan-Meier curves for patients with high (n = 261) and low (n =86) ICPS in the IMvigor210 cohort. (B) Rate of clinical response (complete response (CR)/ partial response (PR) and stable disease (SD)/progressive disease (PD)) to anti-PD-L1 immunotherapy in high or low ICPS groups in the IMvigor210 cohort. (C) Rate of clinical response to anti-PD-L1 immunotherapy in high or low ICPS groups in the IMvigor210 cohort. (D, E) Distribution of ICPS in groups with different anti-PD-L1 clinical response statuses. (F) ROC curve measuring the predictive value of the ICPS. (G) Kaplan-Meier curves for patients with high (n = 23) and low (n =4) ICPS in the GSE78220 cohort. (H) Rate of clinical response (complete response (CR)/ partial response (PR) and stable disease (SD)/progressive disease (PD)) to anti-PD-1 immunotherapy in high or low ICPS groups in the GSE78220 cohort. (I) Rate of clinical response to anti-PD-1 immunotherapy in high or low ICPS groups in the GSE78220 cohort. (J, K) Distribution of ICPS in groups with different anti-PD-1 clinical response statuses. (L) ROC curve measuring the predictive value of the ICPS.

Immunotherapies, such as PD-L1 and PD-1 blockade, have undoubtedly made significant advances in tumor treatments. The low-risk subgroup of patients (n = 86) exhibited longer survival (**Figure 7A**, **Table S13**, *P* = 0.11) compared to the ICPS high-risk subgroup (n = 261) in IMvigor210. Furthermore, the study explored the predictive value of the ICPS in anti-PD-L1 immunotherapy** (Figure 7B-E)**. Patients with ICPS-high risk were more likely to benefit from anti-PD-L1 treatment (**Figure 7B-C**), as confirmed by the Wilcox test (P = 0.029, **Figure 7D-E**). ICPS was identified as a predictive biomarker for anti-PD-L1 immunotherapy benefits (**Figure 7F, AUC = 0.600**). Furthermore, we investigated whether ICPS could play a role in the response to anti-PD-1 treatment using cohort GSE78220. Patients with ICPS-low risk showed better survival (P = 0.19, **Figure 7G)**. Patients with ICPS-low risk showed a better response to anti-PD-1 immunotherapy (**Figure 7H-I**), as indicated by the results of the Wilcox test (P = 0.96, **Figure 7J-K**). The ICPS was further demonstrated to be a reliable predictive tool for the benefits of anti-PD-1 therapy (AUC = 0.538, **Figure 7L**) (**Table S13**). Despite the limited sample size and its non-LUAD origin, the results confirmed that ICPS plays a significant role in predicting the response to immunotherapy.

### Novel Candidate Drugs Treating LUAD

**Figure 8:**
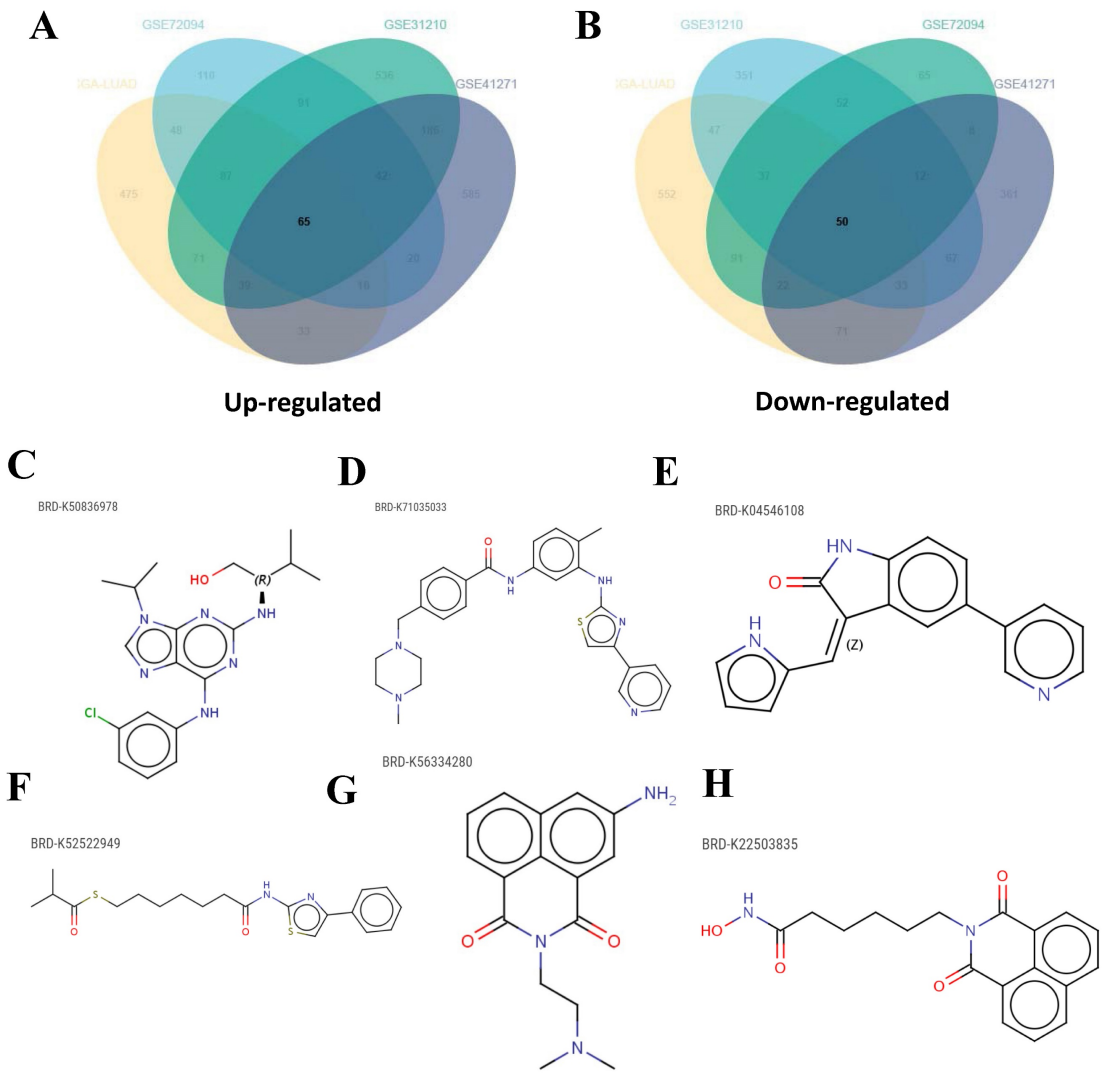
Candidate drugs targeting ICPS identification. (A, B) DEGs identification among ICPS-risk groups. (C-H) The top six drugs that could potentially be used to treat LUAD.

After categorizing LUAD patients into ICPS high and low-risk groups, a total of 115 DEGs consisting of 65 up-regulated DEGs and 50 down-regulated DEGs were identified through a meta-entire cohort (**Figure 8A-B, Table S14**). The top 50 DEGs were then selected for CMap (Connectivity map) analysis. This pattern of gene regulation highly overlaps with several drugs that could be involved in the treatment of PAAD patients (**Table S15**), including BRD-K50836978 (purvalanol-a), BRD-K71035033 (masitinib), BRD-K04546108 (JAK3-inhibitor-VI), BRD-K52522949 (NCH-51), BRD-K56334280 (amonafide), and BRD-K22503835 (scriptaid) (**Figure 8C-H**).

## Discussion

Currently, the incidence and mortality of lung cancer are at the forefront of all kinds of malignant tumors. The most common histological type is LUAD. Historically, surgery, radiotherapy, and chemotherapy have been the primary treatment modalities. Recently, EGFR-TKIs targeted therapy and immunotherapy have emerged as promising approaches for patients with LUAD 43. However, despite having high PD-L1 expression, patients with LUAD did not derive notable benefits from immunotherapy, possibly due to their unique TME 44. TME is composed of tumor cells, stromal components, and immune components. Numerous studies have increasingly shown the significance of immune cell infiltration within the TME in influencing the prognosis of malignant tumor patients and the effectiveness of immunotherapy 45, 46. However, the heterogeneity of tumor patients results in variability in the TME of LUAD patients, potentially contributing to differences in their response to immunotherapy 47, 48. Hence, investigating the heterogeneity of the TME in LUAD patients was deemed crucial for identifying novel strategies in the selection of patients for immunotherapy. In this study, we evaluated the infiltration levels of 24 types of immune cells in 2499 LUAD samples from 14 different public datasets using the ImmuCellAI. ImmuneAI can identify 6 types of immune cells and 18 subsets of T cells, including iTreg, Tc, and exhausted T cells etc. The T cell subpopulations in question are of significant importance in the context of tumor immunity and immunotherapy.

To account for batch effects and errors among multiple platforms, we only considered pairwise comparisons of immune cell infiltration levels within the cohort based on immune cell pairs. Based on the total of 276 immune cell pairs, the ICPS was established using 24 different types of immune cell pairs. We found that the patients with low ICPS showed better OS compared to those with high ICPS. These results demonstrated that the ICPS system was a valuable prognostic indicator for LUAD. What's more, we investigated the correlation between ICPS and immunotherapy. We observed that patients with high ICPS in LUAD had elevated TMB in comparison to those with low ICPS. It has been previously reported that TMB is linked to improved clinical response to single immunotherapy in certain solid tumors, with patients having high TMB exhibiting significantly better response compared to those with low TMB 49, 50.

A variety of immune cells are involved in tumor progression and immune regulation, such as regulatory T cells (Tregs), regulatory macrophages (Mregs), NK cells, tolerogenic dendritic cells (tolDCs), and regulatory B cells. Previous studies have shown that immune cells can affect the prognosis and immunotherapy efficacy of cancer patients, including LUAD 51. Patients with ICPS high-risk scores have more various chemokines, including CCL3, CCL4, CXCL9, and CXCL10, which means that tumors in the body will recruit more CD8+ cells 51-53. CD8+ cells are often considered to have anti-tumor effects, but in our study, we found that patients in the high-ICPS risk score group had higher levels of CD8+ T cell infiltration, which is inconsistent with previous prognoses and has piqued our interest 54. In the analysis of immune checkpoint correlation, we found a significant correlation between LAG3 and ICPS, which may explain this phenomenon. LAG3 as the third immune checkpoint is often associated with poorer prognosis and less immunotherapy benefit at higher levels. Although there is an increase in CD8+ T cells in the high-ICPS patient group, there is also increased expression of LAG3 leading to increased tumor immune suppression 55, 56. Previous studies have confirmed that melanoma patients with LAG3+CD8+ immunotype have worse prognosis after immunotherapy. Therefore, it may be easier for patients in the high-ICPS group to benefit from LAG3+ checkpoint inhibitors and further exploration can be conducted in this direction 57. At the same time, patients in the high-level ICPS group have lower ESTIMATE scores indicating higher tumor purity and poorer prognosis 58.

We evaluated the predictive ability of ICPS on immunotherapy responses using anti-PD-L1 and anti-PD-1 immunotherapy. In IMvigor210, the patients with ICPS low-risk had longer survival. Notably, patients classified as ICPS-high risk were more likely to benefit from anti-PD-L1 treatment, suggesting that ICPS could serve as a predictive biomarker for anti-PD-L1 immunotherapy benefits. Similarly, in GSE78220, patients in the ICPS-low-risk set demonstrated better survival when compared to the ICPS-high-risk set. Furthermore, the ICPS-low-risk group exhibited a better response to anti-PD-1 immunotherapy in comparison to the ICPS-high-risk group, indicating that ICPS may also serve as a viable prediction tool for assessing the benefits of anti-PD-1 therapy. In the PD-L1 treatment group, the high-risk group was more likely to benefit from immunotherapy and reach CR/PR. However, the higher risk group had a worse prognosis, which is consistent with the aforementioned conclusions. This suggests that PD-L1 therapy does not fully improve patient outcomes in the high-risk ICPS group. Moreover, considering the significant differences in LAG3 among patients in different groups of ICPS, it may be that ICPS is more meaningful in predicting patients' recovery from LAG3 inhibitor therapy. However, due to the lack of publicly available datasets on immunotherapy for LUAD, we were unable to verify the predictive ability of ICPS in this context. Therefore, our future research will focus on collecting samples to further study and validate this potential.

In malignant tumors, the presence of a distinct subset of tumor cells exhibiting self-renewal and differentiation capabilities has been confirmed by researchers. These cells, characterized by stemness properties, are referred to as cancer stem cells (CSCs) 59. In primary tumors, undifferentiated CSCs are more likely to disseminate and invade compared to normal tumor cells, thereby contributing to cancer progression and poor prognosis among patients 60. Moreover, CSCs also play a critical role in tumor drug resistance 61, 62. Stemness classification can be utilized by researchers to identify novel molecular markers that can guide clinical tumor treatment and prognosis assessment 63. Various models based on mRNAsi have demonstrated the potential of stemness scores as powerful indicators for predicting tumor prognostic and treatment response 64, 65. We observed that patients with ICPS low-risk exhibited lower mRNAsi scores, mDNAsi, EREG-mDNAsi, DMPsi, and ENHsi in this study. These findings suggest that the ICPS high-risk group possessed a higher degree of tumor stemness, with a tendency toward poorly differentiated and more malignant tumor tissues.

DNA can undergo various types of damage as a result of endogenous and exogenous factors. Among these, DNA double-strand break (DSB) damage is the most cytotoxic 66. Under normal circumstances, the body maintains the integrity and stability of the genome by utilizing repair pathways, with homologous recombination (HR) being one of the repair methods for repairing DSBs 67. Numerous studies have demonstrated the association between HR-related genes or proteins and tumor sensitivity to radiotherapy and drugs. As a novel biomarker, HRD plays a significant role in the individualized treatment of tumors 68. In our study, we observed that patients in the ICPS low-risk group showed lower HRD scores and higher HRD expression in comparison to those in the ICPS high-risk group. These findings suggest that ICPS can reflect the level of HRD and may serve as a suitable tool for guiding tumor prognosis, as well as potentially guiding patients toward the use of PARPi (PARP inhibitor).

In addition to immunotherapy, drug therapy, particularly chemotherapy, remains a primary treatment strategy for LUAD. Therefore, our investigation also aimed to identify potential novel therapeutic candidates for LUAD. We identified six drugs, specifically BRD-K50836978 (purvalanol-a), BRD-K71035033 (masitinib), BRD-K04546108 (JAK3-inhibitor-VI), BRD-K52522949 (NCH-51), BRD-K56334280 (amonafide), and BRD-K22503835 (scriptaid). Further analysis revealed that these drugs may share common mechanisms of action, such as inhibition of histone deacetylase (HDAC) and cyclin-dependent kinase (CDK).

This research also has certain limitations. Despite efforts to collect and utilize multiple LUAD cohorts, we were unable to gather external data for verification, and no experiments have been done to confirm our conclusions. Moving forward, our next step involves collecting data from LUAD patients within our hospital and conducting validation studies on the ICPS in the near future. This is highly significant for predicting the future prognosis and treatment of LUAD patients, particularly in immunotherapy. It also offers insights for developing precise treatment plans.

## Conclusion

In this study, we devised and validated an ICPS as a prognostic indicator for LUAD. This score holds potential as a valuable tool for identifying patients who are suitable candidates for immunotherapy. Our comprehensive assessment of immune cell interactions in LUAD contributes to a deeper understanding of infiltration patterns and functions, thereby guiding the development of more efficacious immunotherapy strategies.

## Supplementary Material

Supplementary figures and tables.

## Abbreviations

LUAD: Lung adenocarcinoma
TME: tumor microenvironment
ICP: immune cell pair
ICPS: immune cell pair score
TCGA: The Cancer Genome Atlas
GEO: gene expression omnibus
ImmuCellAI: Immune Cell Abundance Identifier
ICPI: immune cell pair index
ROC: receiver operating characteristic
SNV: single-nucleotide variant
TMB: tumor mutation burden
mRNAsi: transcriptomic signatures based-index
mDNAsi: DNA methylation based-index
EREG-mDNAsi: epigenetic regulation based-index
DMPsi: differentially methylated probes-based stemness index
ENHsi: enhancer-based stemness index
HRD: homologous recombination deficit
TIDE: T cell dysfunction and exclusion
ICS: immune checkpoints
ICD: immunogenic cell death
DEGs: differentially expressed genes
CMap: Connectivity map
Tcm: central memory T cell
Tem: effector memory T cell
Tr1: tregulatory1 cell
OS: overall survival
nTreg: natural Treg cell
MAIT: mucosal-associated invariant T cell
NK: natural killer cell
CAFs: cancer-associated fibroblasts
Tregs: regulatory T cells
Mregs: regulatory macrophages
tolDCs: tolerogenic dendritic cells
CSCs: cancer stem cells
DSB: DNA double-strand break
HDAC: inhibition of histone deacetylase
CDK: cyclin-dependent kinase
