# Supplementary material for: Leveraging an immune cell signature to improve the survival and immunotherapy response of lung adenocarcinoma
Source: J Cancer. 2024 Jan 1;15(3):747–63. doi: 10.7150/jca.90515 (PMC10777034; doi:10.7150/jca.90515)
Supplement: Supplementary file 1 — Supplementary figures and tables. [file jcav15p0747s1.zip › supplementary legends.pdf]

Supplementary Materials:
Table S1: Basic information of 14 included LUAD cohorts
Table S2: The normalized enrichment scores for the 24 immune cell types among 2499 LUAD patients.
Table S3: Univariate Cox analysis for the 24 immune cell types.
Table S4: Details about the Immune cell pairs (ICPs).
Table S5: ICPs identified by Lasso.
Table S6: Model information about the ICPS.
Table S7: ICPS of 2499 LUAD patients.
Table S8: ICPS and clinical information for the 2499 LUAD patients.
Table S9: ICP score and Gene mutation in GEO databases
Table S10: ICP score and relevant indicators in TCGA-LUAD
Table S11: ICP score and cellular infiltration in TCGA-LUAD
Table S12: ICP score and marker gene in TCGA-LUAD
Table S13: ICP score in IMVigor210 and GSE78820
Table S14: DEGs between high ICPS group and low ICPS group
Table S15: Potential drugs using cMAP.

Figure S1: Difference in prognosis in the median group.
Figure S2: Correlation between ICPS and immune features. (A) Correlation between ICPS and immune cells based on CIBERSORT. (B) Correlation between ICPS and immune cells based on MCPcounter.
