# Supplementary figures and images for: Leveraging an immune cell signature to improve the survival and immunotherapy response of lung adenocarcinoma
Source: J Cancer. 2024 Jan 1;15(3):747–63. doi: 10.7150/jca.90515 (PMC10777034; doi:10.7150/jca.90515)

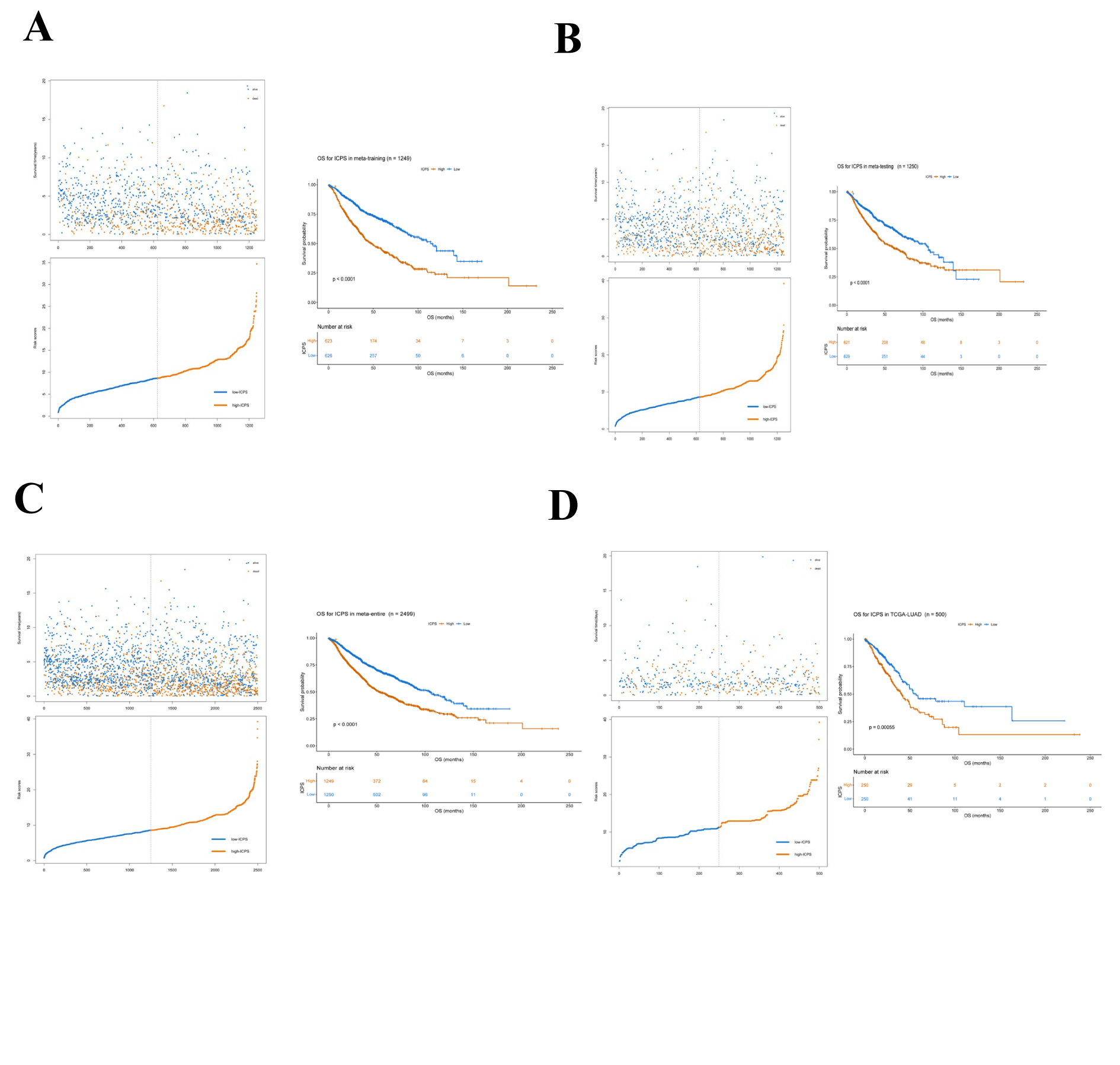

Supplement: Supplementary file 1 — Supplementary figures and tables. [file jcav15p0747s1.zip › Supplementary materals/FigureS1.tif]

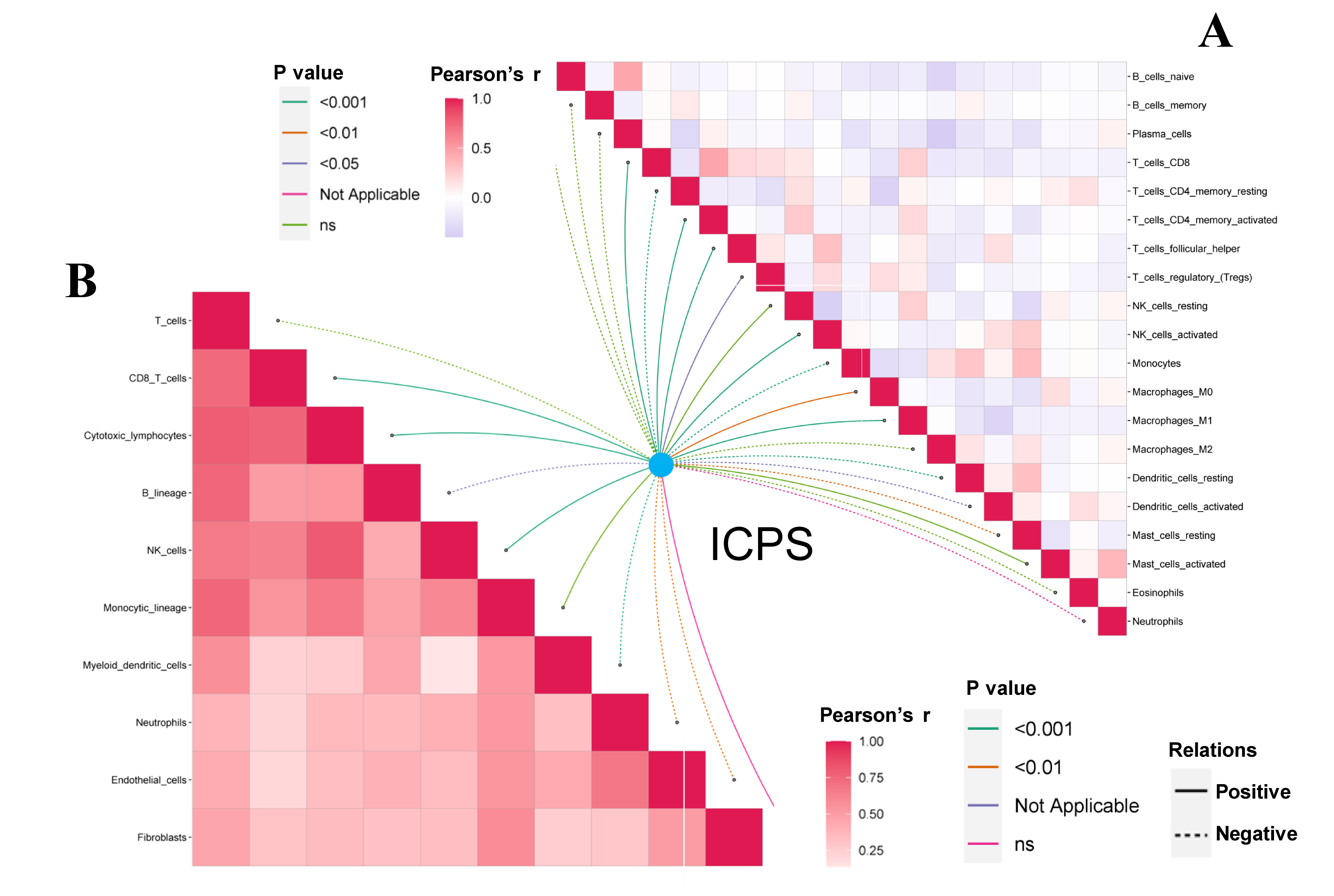

Supplement: Supplementary file 1 — Supplementary figures and tables. [file jcav15p0747s1.zip › Supplementary materals/FigureS2.tif]

A

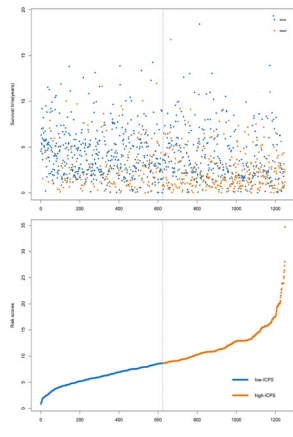

B

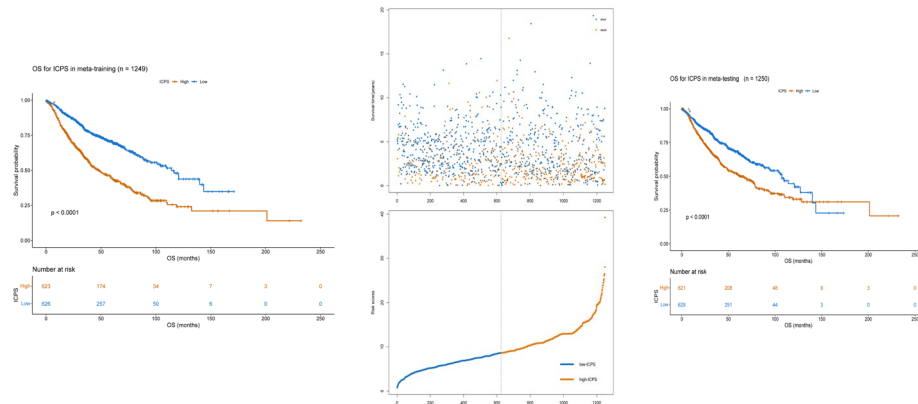

C

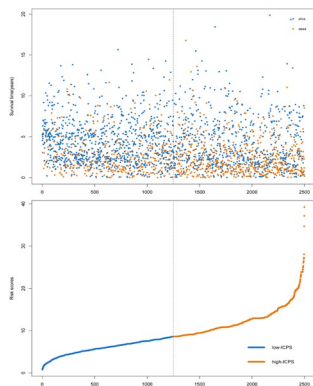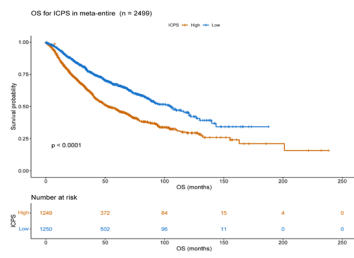

D

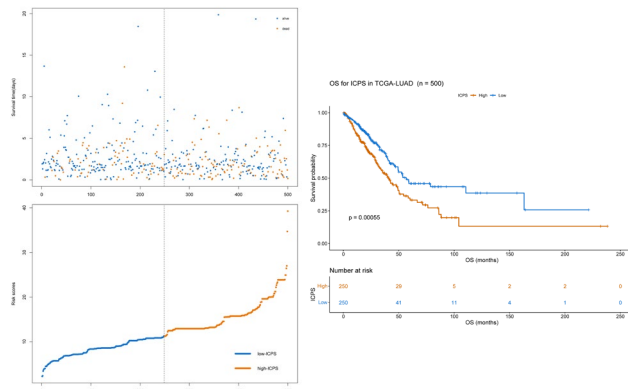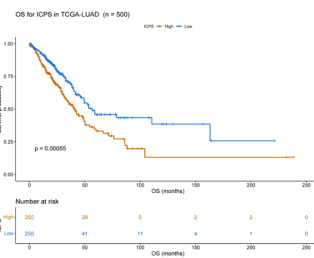

A

P value

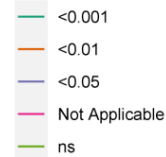

Pearson's r

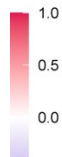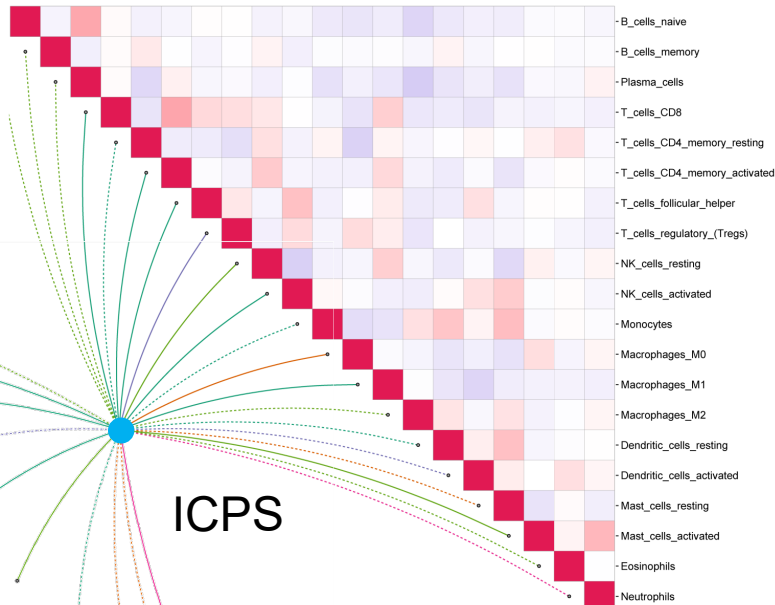

ICPS

P value

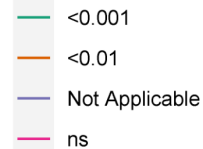

Relations

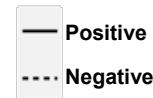

Pearson's r

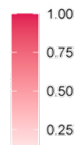

B

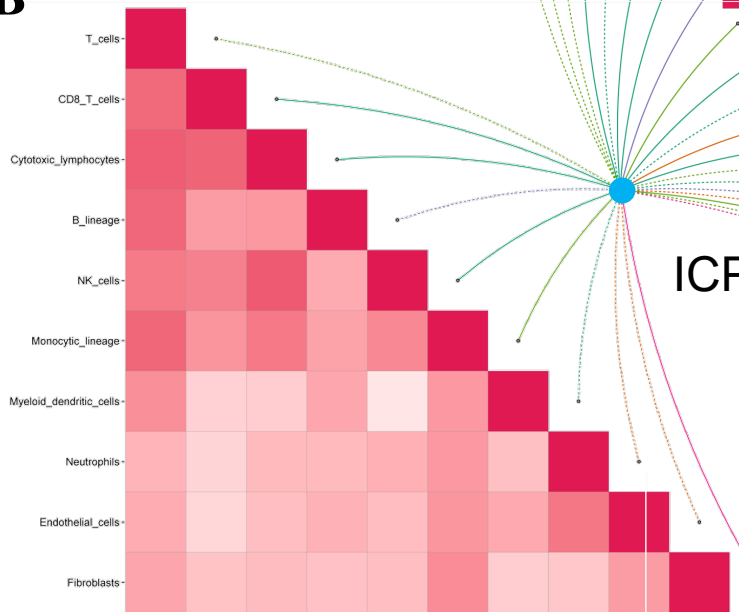

Supplement: Supplementary file 1 — Supplementary figures and tables. [file jcav15p0747s1.zip › Supplementary materals/Supplementary figure.pdf]
